# Cathode Active Material Recycling from Spent Lithium Batteries: A Green (Circular) Approach Based on Deep Eutectic Solvents

**DOI:** 10.1002/cssc.202102080

**Published:** 2021-12-23

**Authors:** Riccardo Morina, Daniele Callegari, Daniele Merli, Giancarla Alberti, Piercarlo Mustarelli, Eliana Quartarone

**Affiliations:** ^1^ Dipartimento di Chimica Università degli Studi di Pavia Via Taramelli 12 27100 Pavia Italy; ^2^ Dipartimento di Scienza dei Materiali Università degli Studi di Milano Bicocca Via Cozzi 55 20126 Milano Italy; ^3^ GISEL-INSTM Via Giusti 5 50121 Firenze Italy

**Keywords:** batteries, deep eutectic solvents, lithium, recycling, soft metallurgy

## Abstract

The transition to a circular economy vision must handle the increasing request of metals required to satisfy the battery industry; this can be obtained by recycling and feeding back secondary raw materials recovered through proper waste management. Here, a novel and green proof‐of‐concept was developed, based on deep eutectic solvents (DESs) to fully and easily recover valuable metals from various cathode active materials, including LiMn_2_O_4_, LiNi_0.5_Mn_1.5_O_4_, and LiNi_0.8_Co_0.2_O_2_. DES composed of choline chloride and lactic acid could leach Li, Mn, Co, and Ni, achieving efficiency of 100 % under much milder conditions with respect to the previous literature. For the first time, to our best knowledge, a two‐step approach was reported in the case of LiNi_0.8_Co_0.2_O_2_ for selective recovery of Li, Co, and Ni with high yield and purity. Furthermore, other cathode components, namely aluminum current collector and binder, were found to be not dissolved by the proposed DES, thus making a simple separation from the active material possible. Finally, this strategy was designed to easily regenerate and reuse the leaching solvents for more than one extraction, thus further boosting process sustainability.

## Graphical abstract

**Metal recycling**: A deep eutectic solvent (DES) consisting of choline chloride and lactic acid extracts Li, Mn, Ni, and Co from cathodes in short time and at low temperature. For LiNi_0.8_Co_0.2_O_2_, the metals are recovered as valuable precursors by using only DES and oxalic acid as chemicals. The leaching DES is regenerated and employed for multiple extractions.

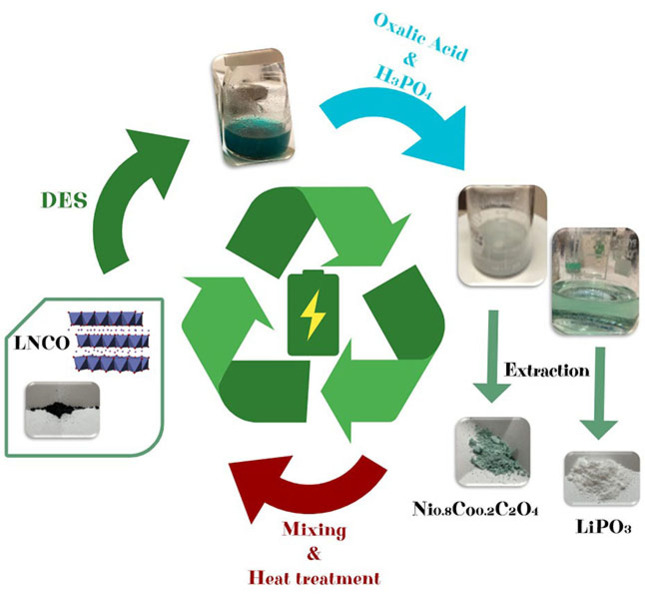

## Introduction

Raw materials (RMs) supply is essential for every industrial chain and constitutes one of the bases of global growth. The rapid development of technologically innovative scenarios (e. g., electric mobility, Internet of Things, IoT, etc.) increases the demand for metals and minerals, which will double in the next ten years.^[1]^ Especially in the case of the European Union (EU), there is a strong imbalance between RMs demand and their supply, which is limited by the scarcity of mines. Consequently, the RM value chain is not fully covered by the EU industry, and this aspect is crucial for the survival of the EU economy itself. To guarantee secure access to these valuable products, the European Commission (EC) has established a list of the critical raw materials (CRMs),[^1^, ^2^] which is relevant for many high‐tech applications, including low/zero‐carbon technologies (photovoltaics, wind turbines, batteries, fuel cells), the production of which will increase the CRM demand by a factor of about 20 by 2030. Si, Ge, Co, and Pt‐group metals are just a few examples of elements essential for developing green energy applications.^[1]^

Batteries are a key enabling technology for the integration of renewables into the grid and zero‐emission electric mobility. The demand for batteries is intended to grow exponentially in the near future, corresponding to a global battery production of about 500 GWh by 2025.[^3^, ^4^] In particular, lithium‐ion batteries (LIBs) are the current technology on which electric mobility is based.^[5]^ A LIB is embedded in different CRMs of high economic importance and supply risks depending on the cell chemistry. The essential RMs for battery production are Co, Li, natural graphite (NG), Si, Ni, and Mn. At present, at least three are considered critical by EC, namely Si, Co (vulnerable to supply chain interruptions), and NG. However, also the other ones are expected to become critical in the next decades. Their primary sources are not present in the EU and are placed mainly in four countries: China (Si, NG), Congo (Co), South Africa (Mn), and Chile (Li, Cu).^[2]^

To satisfy the battery industry demand, a large increase of metals will be required (3 times for Co and 3.5 for Li) before 2025.^[2]^ This can be supported by the transition to a circular economy vision, through recycling and feeding back secondary RMs recovered through proper waste management.[^1^, ^2^] Economic analyses based on different scenarios proved that achieving high levels of batteries recycling will have strong benefits in terms of dependence on imported materials, environment, and employment.^[6]^ To this aim, the EU Battery Directive put in place obligations for the member States and industry actors to maximize spent batteries collection and to set up proper recycling treatments.^[7]^ The consequence was the development of several EU projects on circular economy and reuse of batteries (e. g., Crocodile, ColaBatts) and the increasing investment from some companies to recycle exhausted EV batteries.^[8]^ In January 2021, Volkswagen Group Components opened a battery recycling pilot plant in Salzgitter, able to process 3600 battery packs per year by an innovative and CO_2_‐saving process, contributing to making the e‐mobility concretely zero‐emission.^[9]^ Overseas, in May 2021 Ultium Cells LLC, a joint venture between General Motors and LG Chem, announced an agreement with Canadian Company Li Cycle to recycle up to 100 % of the battery critical materials from the scrap generated by its Lordstown plant, a mega‐factory in Ohio (www.li‐Cycle.com).

As stated, current LIBs consist of cathodes containing Li and transition metals (e. g., Ni, Mn, Co, in short: NMC) and a graphite anode. Spent LIBs are considered hazardous waste due to the presence of toxic oxides such as LiCoO_2_, LiMn_2_O_4_, LiNiO_2_, and others. They usually contain 5–20 % Co, 5–10 % Ni, 5–7 % Li, 5–10 % other metals (Cu, Al, Fe, etc.), 15 % organic compounds, and 7 % plastic.[^10^, ^11^] The metallurgy processes to remove metals from wastes need to fit the complexity of the batteries and the electronic nature of the waste. They must be also able to separate metals from other components, such as polymers. Several good reviews describe in detail the entire recycling process of end‐of‐life batteries, which consists of several steps, including assessment, diagnostics, pack and module disassembly, stabilization and discharging before of the physical separation of the module components, and finally recovery of the cell materials, depending on its chemistry.[^12^, ^13^, ^14^, ^15^] This last point is critical because of two main aspects: (i) process optimization to produce usable materials streams, and (ii) process sustainability in terms of cost and environmental footprint.^[16]^

The state‐of‐the‐art recovery methods employed for metal (Co, Li, Ni) extraction are pyrometallurgy (using high‐temperature furnaces to reduce the metal oxide components to metal alloys) and hydrometallurgy. The latter approach is based on the treatment of the black powder coming from the mechanical treatment of the dismantled batteries to recover metals by leaching with mineral acids and reducing agents and subsequent precipitation in the salt form.^[12]^ However, hydrometallurgical processes are highly energy‐consuming, require harsh conditions, and produce toxic materials, thus resulting in drastic environmental drawbacks.[^10^, ^17^] While they are currently used in industrial plants (e. g., by Umicore, Recupyl, or Duesenfeld), greener routes need to be explored to recover CRMs in the context of sustainable circular economy approaches.^[12]^ Among them, soft solvometallurgy, based on deep eutectic solvents (DESs), is gaining attention due to the combination of good recovery efficiency, low cost, biodegradability, and, in some cases, ability to dissolve metal oxides.[^18^, ^19^, ^20^]

DES are an emerging class of green solvents based on binary or ternary mixtures with huge melting point depression at the eutectic composition compared to those of the components. They are formed by mixing hydrogen bond donors (HBD; e. g., urea, glycerol, carboxylic acids, acetamide, etc.) and hydrogen bond acceptors [HBA; typically choline chloride (ChCl) or other quaternary ammonium chlorides].^[20]^

The suitability of DES as leaching media to recover Li and Co from the cathode powders of LIBs was recently demonstrated starting from LiCoO_2_,[^21^, ^22^, ^23^] where ChCl mixed with ethylene glycol, citric acid, and urea gave extraction yields higher than 90 %. Good DES selectivity was also proved by studying the solvent extraction fractionation in the case of more complex matrices as NMC cathodes.^[24]^ Such a process was based on the use of *N*,*N*,*N*′,*N’*‐tetra‐*n*‐octyldiglycolamide (TODGA) diluted in an imidazolium‐based ionic liquid. Mn was extracted in a single step with 99 % efficiency, followed by the extraction of Co (>90 %) by alkyl phosphonium chloride and finally of Ni and Li via a DES, based on lidocaine and oxalic acid.

Despite such promising results, the use of DESs still has some issues: (i) the higher temperature (>170 °C) and longer times (>24 h) of treatment compared to mineral acids like HNO_3_ and HCl, required to obtain high leaching yield, and (ii) the complexity of chemical processes needed to recover the metals from precipitation, mostly in case of Li, as valuable products or precursors for the re‐synthesis of cathode materials.

This article reports on a highly solvometallurgical approach to recover critical raw elements, namely Li, Co, Ni, and Mn, from several types of cathodes materials for LIBs, including LiMn_2_O_4_ (LMO), LiNi_0.8_Co_0.2_O_2_ (LNCO), and LiNi_0.5_Mn_1.5_O_4_ (LNMO). A ChCl–lactic acid (ChCl:LA) mixture was optimized to successfully leach metals with high yield at low temperature and short times and recover all of them as the starting precursors or oxides for cathode production. In the specific case of LNCO, this DES was also investigated for a potential reuse in multiple extractions to reduce the amounts of reactants, thus further enhancing the overall sustainability of the recycling process.

## Results and Discussion

### DES selection for the leaching step: Influence of time, temperature, and solid loading

Like ionic liquids (ILs), DESs have low volatility, tunable polarity, and high thermal stability. With respect to ILs, they are easier to prepare, less expensive, more biodegradable, and safer.^[20]^ Furthermore, in the case of ChCl as a mixture component, it was demonstrated that they are capable of dissolving several metal oxides due to the presence of a strong coordinating anion for the complexation of the metal oxide to form soluble species.^[19]^ As stated, two DESs were prepared, namely ChCl:LA and ChCl‐glycerol (ChCl:Gly)?, in molar ratio 2 : 1. At this molar composition both the systems were liquid at room temperature, with melting temperature well below −70 °C, as evidenced by the absence of exotherms/endotherms in the cooling/heating differential scanning calorimetry (DSC) thermograms reported in Figure S1.

### LiMn_2_O_4_ and LiNi_0.5_Mn_1.5_O_4_

**Figure 1:**
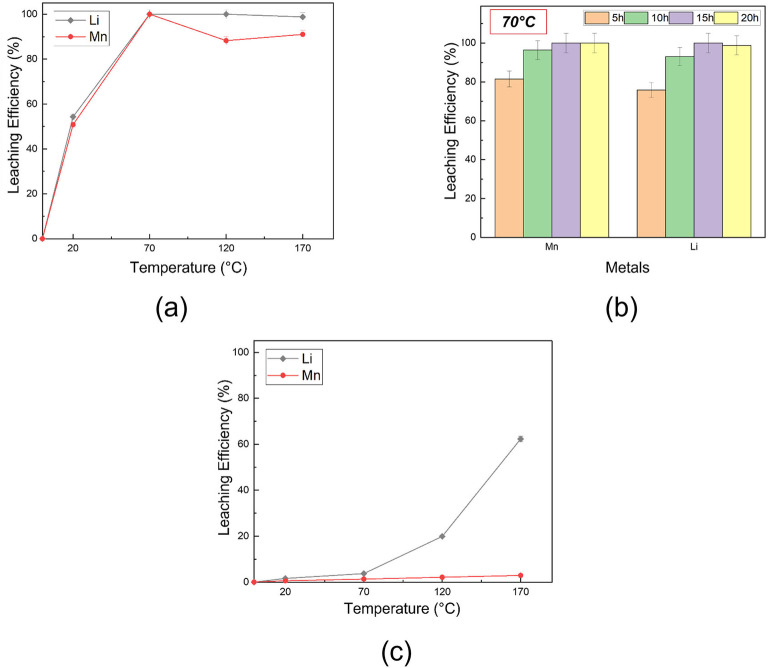
Leaching efficiency of the investigated DES in case of LiMn_2_O_4_: (a) ChCl:LA vs. temperature (*t*=24 h); ChCl:LA vs. time (at 70 °C); (c) ChCl:Gly vs. temperature (*t*=24 h). If the error bars are not visible, the standard deviation is less or equal than the symbol dimension.

The leaching procedures were first carried out on LMO by investigating both temperature and time as the variables. Specifically, Li and Mn extraction was carried out by dispersing the oxide in the different DES, as described in detail in the Experimental Section, and tested at four temperatures, namely 20, 70, 120, and 170 °C for 24 h, or at 70 °C for 5, 10, 15, and 24 h. Figure 1 shows the leaching efficiency obtained from inductively coupled plasma optical emission spectroscopy (ICP–OES) analysis performed on ChCl:LA (Figure 1a, b), and ChCl:Gly‐based solutions (Figure 1c) at different temperatures and times. The results indicated that the system ChCl:LA is more efficient than ChCl:Gly in dissolving the selected cathode.

Indeed, ChCl:LA extracted more than half of the metal content at 20 °C after 24 h. A heating treatment at a relatively low temperature of 70 °C is enough to reach nearly 100 % of Li leaching efficiency. In contrast, the Mn data are more scattered due to higher uncertainty of the ICP measurements (≈10 %), and a slightly lower (≈90 %) leaching yield was obtained across the examined temperature range. However, both Li and Mn could be almost fully extracted after 10 h at 70 °C (Figure 1b). Compared to other DES‐based metal recovery processes discussed in the literature,^[21]^ where higher temperature and longer times were required to achieve full metal leaching, our results are better in terms of sustainability and energy saving. ChCl:Gly was remarkably less efficient in the metal extraction, as shown in Figure 1c. In fact, only partial dissolution of Li was obtained after 24 h of heating at 170 °C with a yield of about 60 %, whereas negligible leaching (<5 %) was obtained for Mn. This behavior may be interpreted in terms of different pH and HBD properties. In fact, ChCl:LA showed lower pH (0.4) than ChCl:Gly (3.9). In addition, LA has better complexing properties than Gly, owing to the greater ability of H^+^ to act as an oxygen acceptor. The combination of such two properties was found to greatly increase the solubility of metal oxide.^[25]^

**Figure 2:**
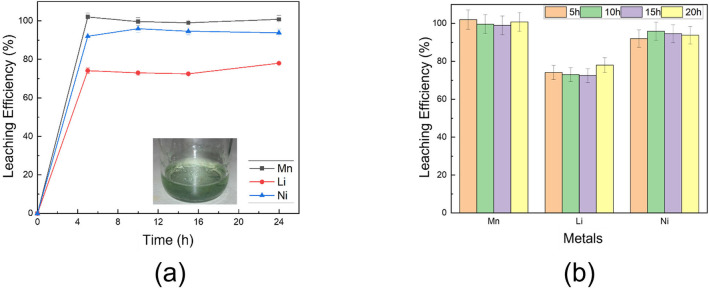
Leaching efficiency of the ChCl:LA in case of LiNi_0.5_Mn_1.5_O_4_: (a) vs. time and (b) vs. metal.

On this basis, ChCl:LA was selected for further analyses. Another LMO‐based active material was tested, namely LNMO, to check the efficiency of this DES in the extraction process of different metals. Figure 2 reports the leaching efficiency for Li, Mn, and Ni, this last element in the case of LNMO, as a function of time. A treatment as short as 5 h at 100 °C was enough to extract almost the Mn (≈100 %) and Ni (≈95 %) fraction, but not optimal in case of Li, for which a leaching efficiency of 75 % was obtained. Several reasons affecting the dissolution metal yield were deeply discussed in the literature, such as the ionic activity, reaction rates, electron transfer coefficient, and diffusion processes, which are affected by both temperature and time (see, e. g., Ref. [23]). In this specific case, a slight partial precipitation of Li salts cannot be excluded to rationalize lower Li extraction yields. Indeed, fine crystals were sometimes observed in the leached solution upon long standing and storage.

### LiNi_0.8_Co_0.2_

LNCO was also tested due to the presence of Co, which is likely the most critical raw element in LIBs and likely the more complex to recover. More or less intensely blue‐colored solutions were obtained, depending on the solid loading, which is typical of the (CoCl_4_)^2−^ complex deriving from the cathodic active materials dissolved in the presence of ChCl.^[22]^ First attempts to dissolve and recover LNCO through the same conditions used in case of LMO and LNMO were not fully satisfactory. Consequently, to obtain more information on the influence of the experimental parameters, a more systematic approach was applied based on design of experiment (DoE),^[26]^ a chemometric tool allowing to optimize processes with relatively few experiments, highlighting the optimal conditions that may not be obvious at the beginning of the study. The DoE was carried out starting from the ICP analysis performed on the leaching solutions, as summarized in Table 1 and Table S1 in the Supporting Information.

**Table 1:** Leaching efficiency (L.E.) from quantitative chemical analysis of Li, Co, and Ni obtained by ICP‐OES measurements used for DoE in LNCO.

Experiment	*T*	*t*	DES	Metal L.E. [%]		
	[°C]	[h]	[g]	Co	Li	Ni
1	50	5	2.5	33.0	40.6	30.7
2	105	5	2.5	87.8	72.4	86.5
3	50	24	2.5	51.5	58.5	51.1
4	105	24	2.5	88.2	74.0	87.5
5	50	5	7.5	62.4	62.4	62.1
6	105	5	7.5	100	100	100
7	50	24	7.5	70.6	68.1	70.8
8	105	24	7.5	100	100	100
9	77.5	14.5	5.0	64.7	67.3	64.9

The variables that may reasonably influence the metal recovery percentage are temperature (*T*), extraction time (*t*), and quantity of DES (*m*_DES_). The variables extremes, as reported in Table 2. These extremes limits were chosen in such a way as to make the process economically sustainable and favor industrial scalability. Preliminary data show linear trends outside the ranges considered.

**Table 2:** Levels of the experimental domain.

Variable	*T* [°C]	*T* [h]	Quantity of DES [g]
−1	50	5	2.5
+1	105	24	7.5

On this basis, a full factorial 2^3^ DoE was applied. Table 3 reports as an example the design of experiments and the experimental plan obtained in the case of Co. For each experiment, two replicates were performed. The data regarding Li and Ni analysis are reported in the Supporting Information as Figure S2.

**Table 3:** DoE, experimental plan, and the experimental response obtained from the cobalt extraction by ChCl:LA DES.

No.	2^3^ experimental design			Experimental plan			Response 1st replicate	Response 2nd replicate
	*T*	*t*	*m*_DES_	*T* [°C]	*T* [h]	*m*_DES_ [g]	Co recovery [%]	Co recovery [%]
1	−1	−1	−1	50	5	2.5	33.0	37.6
2	1	−1	−1	105	5	2.5	87.8	79.5
3	−1	1	−1	50	24	2.5	51.5	52.2
4	1	1	−1	105	24	2.5	88.2	85.0
5	−1	−1	1	50	5	7.5	62.4	58.3
6	1	−1	1	105	5	7.5	105.9	100.0
7	−1	1	1	50	24	7.5	70.6	71.0
8	1	1	1	105	24	7.5	100.1	88.1

For the model, Equation (1) was applied:

(1) $$R=b{}_{0}+b{}_{1\times 1}+b{}_{2\times 2}+b{}_{3\times 3}+b{}_{12\times 1}x{}_{2}+b{}_{13\times 1}x{}_{3}+b{}_{23\times 2}x{}_{3}$$

Where R is the response, b the regression coefficients, *x*_1_ is the temperature (*T*), *x*_2_ the time (*t*), and *x*_3_ the quantity of DES (*m*_DES_).

For DoE calculations, the open‐source program CAT (Chemometric Agile Tool)^[27]^ was used.

**Figure 3:**
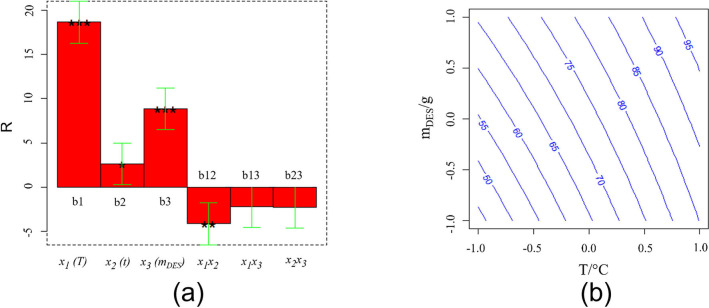
(a) Values of the coefficients obtained from the multi regression of Equation (1) for the 2^3^ full factorial design, performed to optimize cobalt extraction by DES. (b) Iso‐response curve *m*_DES_ vs. *T*.

Figure 3a shows the plot of the model coefficients; asterisks indicate their significance according to the usual convention: *=*p*<0.05, **=*p*<0.01, ***=*p*<0.001. The coefficient sign indicates in which direction each variable has to be set to increase the response.

Similar curves were obtained in the case of Ni and Li, as shown in Figure S2a–c. For each metal, *T*, *m*_DES_, and their interactions (*x*_1×3_) have a major effect on the response, whereas the variable *x*_2_, namely the extraction time, is less significant. Therefore, such two parameters, *x*_1_ and *x*_2_, have to be set at their high level to maximize the response (Li, Co, and Ni extraction [%]). The results can be usefully represented as iso‐response curves, reported in Figure 3b, the axes of which refer to the codified variables *T* and *m*_DES_, with the third variable, *t*, kept at 0 codified value since this last variable is always less significant than the other two. From this graph, it is evident that the highest percentage of recovered Li (and similarly of Ni and Co) is obtained at a temperature of 105 °C and using an amount of DES of 7.5 g (corresponding to a solid loading of 16 g L^−1^), independent of the extraction time.

### Metal recovery after leaching

#### Co and Ni recovery

**Figure 4:**
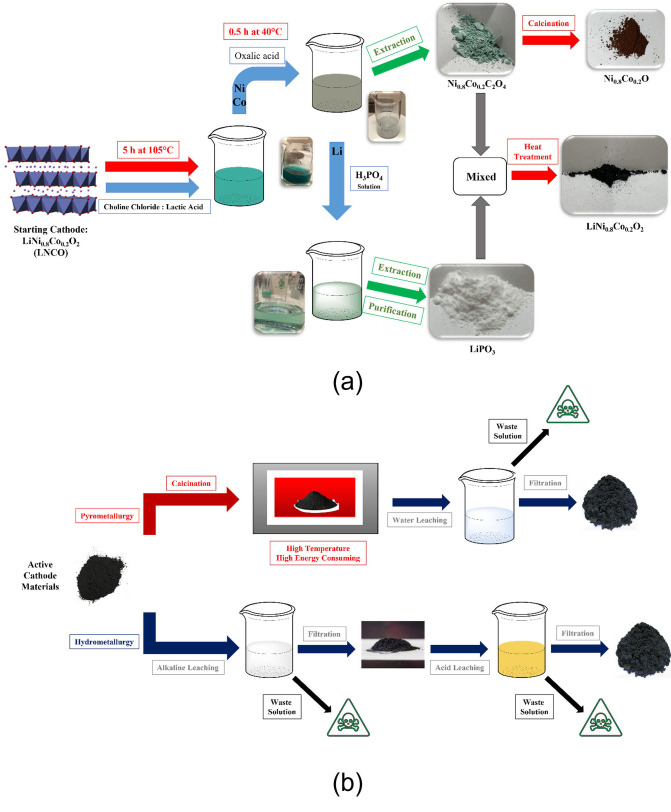
(a) Proof‐of‐concept for DES‐based recycling loop from LNCO cathode material. (b) Scheme showing pyrometallurgy and hydrometallurgy methods typically used to recover metals from LIBs.

The procedure to recover Li, Ni, and Co from the LNCO leaching solution and the subsequent steps to regain the active materials are depicted in Figure 4a. A visual comparison including a conventional hydrometallurgical process (typically based on mineral acids and strongly reducing reactants) is also reported in Figure 4b to further highlight the sustainability of the proposed strategy.

The first step was the precipitation of Co and Ni by using oxalic acid (OA) as the agent. The reason for such a choice is that lithium oxalate is highly soluble in this matrix, whereas Co and Ni oxalates show fast precipitation in ChCl:LA:OA because of the strong interactions of the metal with the oxalate anion. OA is, in fact, highly selective towards complexation and precipitation of metals such as Co, Ni, and Mn, and this property is certainly beneficial in the case of multi‐metal extraction processes from LIBs.^[25]^

OA was used in two ways: (i) in water solution (0.25 m as the saturated solution), and (ii) as a solid component. The second approach was followed to avoid any other solvents than DES, in order to evaluate the feasibility of multiple extractions, as better discussed in the following.

**Figure 5:**
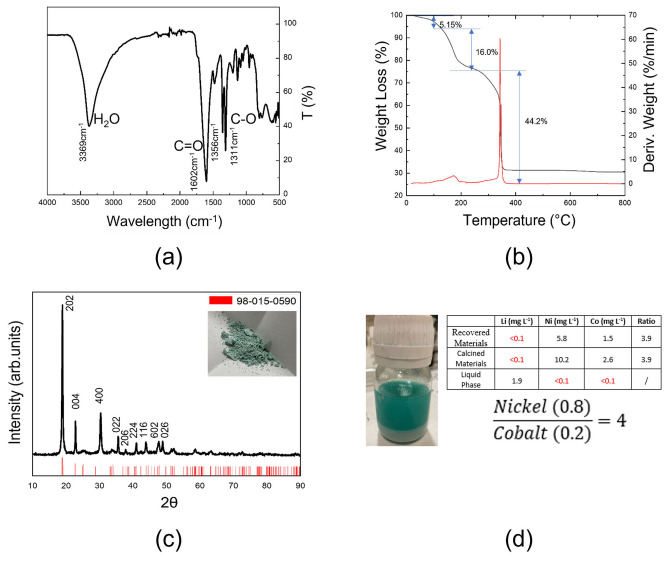
(a) FTIR spectrum; (b) TGA plot; (c) XRD pattern of the material recovered by treating the leaching solution with OA. The red reference lines are referred to the powder file JCPDS 98‐015‐0590. (d) Amount of metals from ICP in the material as recovered, after calcination, and in the leaching solution after treatment with oxalic acid. The standard deviation for each average concentration is always lower than 6 %.

The precipitation treatment was performed under mild conditions in both cases, heating at temperatures lower than 40 °C for short (30 min and 1 h) and longer times (4 h). A light‐green precipitate was separated with comparable yields, independently on the chosen time, which was characterized using ICP‐OES, Fourier‐transform infrared (FTIR) spectroscopy, and X‐ray diffraction (XRD) to determine the metal recovery yield and identify the material phase. Figure 5 shows (a) the FTIR spectrum, (b) the thermogravimetric analysis (TGA) plot, and (c) the XRD pattern of the recovered compound.

Specifically, the FTIR spectrum (Figure 5a) shows an intense band at 3369 cm^−1^, assigned to the O−H bond stretching vibration of water, one peak at 1602 cm^−1^ typical of the carbonyl stretching, and a double signal at 1356 and 1311 cm^−1^, ascribed to the C−O bonds in the oxalate carboxyl groups. The spectrum is in very good agreement with that reported in the National Institute of Standards and Technology (NIST) databases for Ni and Co oxalate (NIST Chemistry Web book, https://webbook.nist.gov/chemistry). The thermogravimetric plot (Figure 5b), obtained during the decomposition of the recovered material under air atmosphere, reveals a two‐step degradation, the first weight change occurring in the temperature range 100–220 °C when the crystallization water was lost, and the second one between 220 and 400 °C, due to the evolution of CO_2_ and consequent formation of the metal oxide. The weight losses associated with both processes (21.1 and 44.2 %, respectively) are in good agreement with that expected if all the water and organics were lost (namely 20 and 41 %, respectively). The difference should be attributed to the presence of impurities in the precipitated material. The salt was obtained with a very high recovery yield (>85 %). According to the thermal results, it is compatible with in the dihydrate form with minimum molecular formula Ni_0.8_Co_0.2_C_2_O_4_ ⋅ 2 H_2_O.

This conclusion is further proved by XRD: the pattern, reported in Figure 5c, is fully aligned to that of Ni and Co oxalate with two crystallization water molecules, according to the powder file JCPDS 98‐015‐0590. The ICP‐OES analyses (see Table in the inset of Figure 5d), carried out on the recovered salt, nicely confirmed the stoichiometry expected from the thermal data, pointing to a Ni/Co ratio of 3.9, in excellent agreement with the nominal one that is 4.0. This result also suggests that the metals underwent precipitation almost simultaneously, as expected considering the similar chemistry of Ni and Co. The quantitative data also evidence the absence of Li in the recovered powder, except for traces (see Table in Figure 5d).

**Figure 6:**
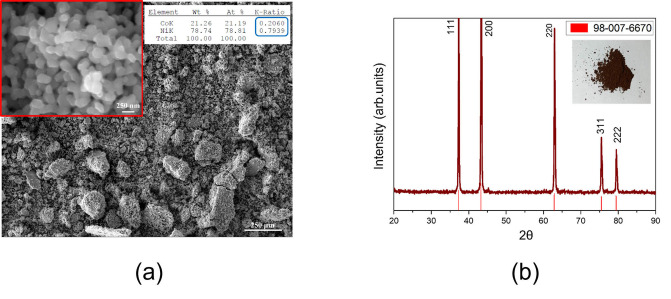
(a) Scanning electron microscopy (SEM) images at different magnifications and EDX results, and (b) XRD pattern of the Ni−Co oxide obtained by calcinating the recovered oxalate. The red lines in the diffractogram are referred to the JCPDS 98‐007‐6670 file.

The subsequent calcination of the mixed Ni−Co oxalate at 70 0 °C in air for 12 h led to a brown powder, whose Ni/Co ratio of 3.9 is very well preserved, as further confirmed by energy‐dispersive X‐ray spectroscopy (EDX) analysis reported in the Table of the inset of Figure 6a. The corresponding XRD pattern in Figure 6b exhibits a highly crystalline and pure phase, the peaks of which match those of rock salt (cubic) NiO (JCPDS 98‐007‐6670). The brown color is typical of the presence of Co in the bulky Ni oxide (known as “nickel brown”). The calcined powder consisted of aggregates of almost uniformly spherical particles, with diameters ranging between 100 and 250 nm (Figure 6a).

The use of OA as a pure solid component rather than in aqueous solution did not affect the precipitation mechanism, similarly, leading to the mixed dihydrate oxalate salt, namely Ni_0.8_Co_0.2_C_2_O_4_  ⋅ 2 H_2_O, and nickel brown after the calcination step. The results are reported in Figure S3. This is important from a practical point of view, since the possibility to avoid water significantly simplifies and speeds up the DES regeneration.

### Recycling of DES: multi‐extraction process

**Figure 7:**
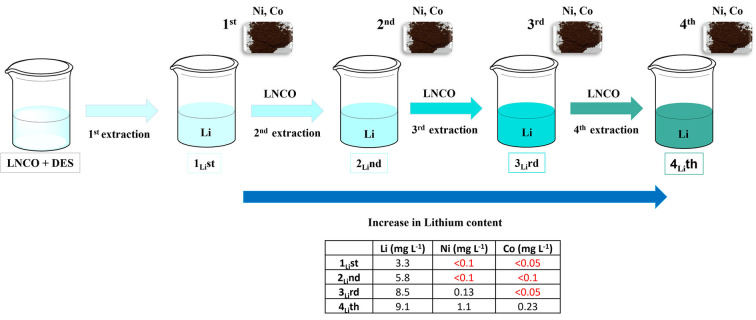
Scheme of multi‐extraction experiments (4 cycles) to evaluate DES recyclability: (i) first step: dissolution of LNCO in DES; (ii) first separation of the three metals: Ni and Co extraction using OA (sample 1st–4th); (iii) restoring the original volume and second addition of LNCO at the same concentration. All these steps were repeated three more times. Li was accumulated in the leaching solution during each cycle up to the end of the process, and the corresponding concentrations (solutions 1_Li_st–4_Li_th) are reported in the Table. ICP measurement errors were below 10 %.

The feasibility of DES recycling to allow use in a multi‐extraction process was also investigated. Four recovering steps were tested, as described in Figure 7. (i) LNCO was first dissolved in DES at 105 °C for 5 h, and the resulting leaching solution was treated with OA to precipitate Ni and Co, accumulating Li in the leaching matrix. After the first separation of the recovered Ni/Co oxalate, the remaining DES solution (sample 1_Li_st) was used for a second extraction by dissolving another amount of LNCO in the same conditions and treating again with OA for a second precipitation of Ni/Co oxalate. (ii) These steps were carried out two more times (samples 3rd and 4th).

**Figure 8:**
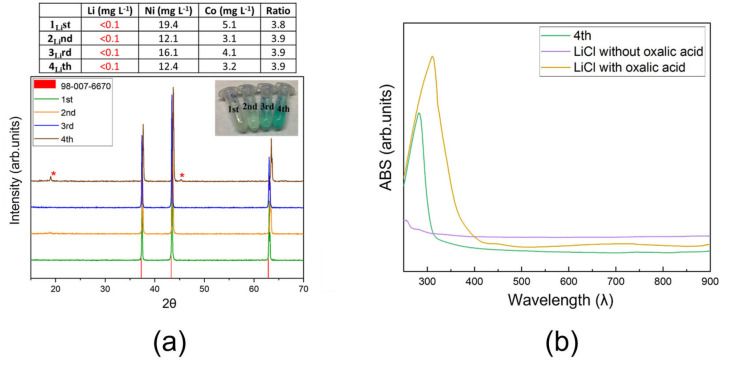
Metal multi‐extractions (number of extracting cycles: 4). (a) XRD patterns of the materials recovered after each cycle. (b) UV/Vis absorption spectra of a reference solution composed by LiCl dissolved in ChCl:LA (purple line), the same reference solution of LiCl in ChCl:LA supplemented with OA, which mimics the Li‐including leaching solution (yellow line), and the real leaching solution after 4 extraction cycles. The errors of the data in the Table were below 5 %.

Each step was followed by ICP‐OES analysis to evaluate the metal separation and the Ni/Co recovery rate during the multi‐extraction. The Table in Figure 7 reports the amounts of Li, Ni, and Co detected in the oxalate salts recovered at each step. The data suggest the DES could be effectively recycled for at least three subsequent extractions, leading to similar Ni and Co recovery yields (higher than 80 %) and Ni/Co ratio. After that, the leaching capability of the recycled solvent started to decrease, as evident in the XRD pattern of the four recovered powders reported in Figure 8a. Contrary to the Ni/Co oxalate obtained with cycles 1–3, where only the diffraction peaks of the salt were present, in the case of the fourth extraction two additional signals appeared at *θ*=19 and 45°, corresponding to the more intense peaks of LNCO, which showed that DES no more fully dissolves the active material.

As evident in the inset of Figure 8a, the color of the recycled DES, successive to the first Ni and Co precipitation, takes on a more and more intense blue/green tone during the following extractions. This phenomenon is an index of Li accumulation during the multi‐extraction cycles. Indeed, the ICP‐OES results, summarized in the Table of Figure 7, show an almost linear increase of the Li‐ion concentration. Such a color suggests the formation of one complex composed of Li as metallic center and chloride and oxalate as anion ligands. This hypothesis is supported by UV/Vis spectroscopy performed comparing the sample 4_Li_th (chosen as a representative example) with two reference solutions with similar molar ratios, namely LiCl in ChCl:LA and LiCl in ChCl:LA:OA (Figure 8b). The LiCl:ChCl:LA system is colorless, as expected; however, in consequence of the OA addition, the solution turns blue/green, and the corresponding UV/Vis spectrum is comparable to that of the sample 4_Li_th with one maximum similarly peaked around 300–325 nm.

In the light of the transfer of such proof of concept to the whole cathode recycling, the compatibility of other electrode components, such as polyvinylidene fluoride (PVDF) as the binder and aluminum foil as the current collector, was also tested. No dissolution phenomena occurred when both the current collector and PVDF were treated with the DES at 105 °C for 5 h (Figure S4). This is important because it allows us to envisage a subsequent step for the mechanical separation of these components.

### Li recovery

Although Ni and (mostly) Co are undoubtedly the most valuable metals in terms of CRMs, Li is also present in a non‐negligible amount, and its recovery may deserve proper focus in view of optimized battery recycling processes. At present, Li recovery is only assumed possible by precipitation via Na_2_CO_3_ aqueous solution.^[21]^ Even though this strategy may be successfully used in conventional hydrometallurgical processes, this does not seem the case for solvometallurgy via ChCl‐based DES. Indeed, Li complexation with Cl^−^ ligands is preferential and strong, and thereby the precipitation of lithium carbonate is unfavored.

**Figure 9:**
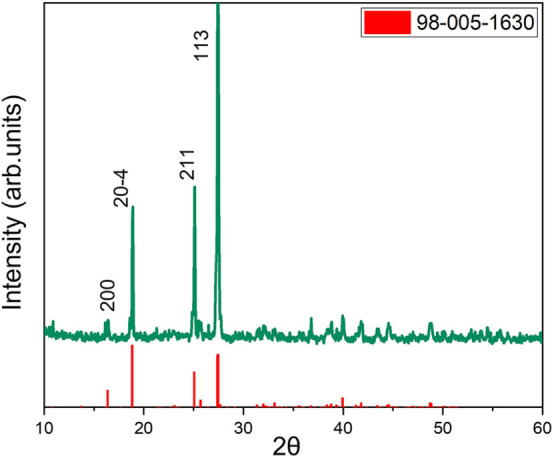
XRD pattern of the material recovered after treating the Li‐containing solution with phosphoric acid. The red lines are referred to the powder files JCPDS 98‐005‐1630, suggesting the formation of LiPO_3_.

After the extraction of Ni and Co, the remaining Li leaching solution was treated with a 0.5 m H_3_PO_4_ solution, as already described in the Experimental Section. A pale grey powder was obtained, which was then separated by centrifugation and finally purified. Figure 9 shows the XRD plot of the recovered material, revealing a highly crystalline system whose pattern well matches the structure of lithium metaphosphate LiPO_3_ (JCPDS 98–005‐1630).

### Synthesis of LNCO from the recovered materials and electrochemical tests

**Figure 10:**
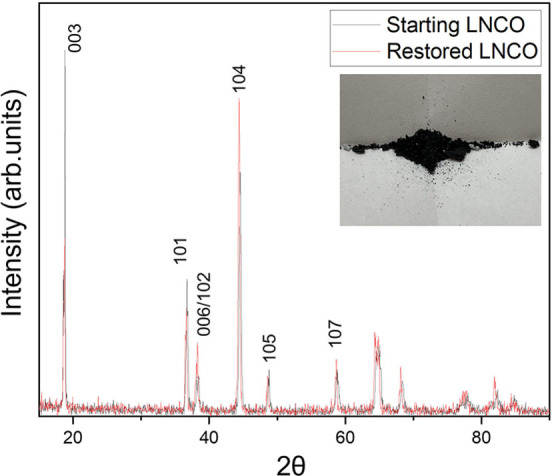
Comparison of the XRD patterns of pristine (black lines) and the recovered (red line) LNCO.

The clear identification of the phases coming from the metal extractions allowed the synthesis of LNCO as a recovered cathode active material. It was prepared using a solid‐state reaction by properly mixing the recovered Ni and Co oxalate and Li metaphosphate in stoichiometric amounts and treating them by a two‐steps thermal process at 550 and subsequently at 750 °C, for 12 h in both cases. XRD demonstrated that the resulting powder was LiNi_0.8_Co_0.2_O_2_, as clearly shown in Figure 10, where the XRD patterns of the starting commercial cathode and the recovered one are compared.

**Figure 11:**
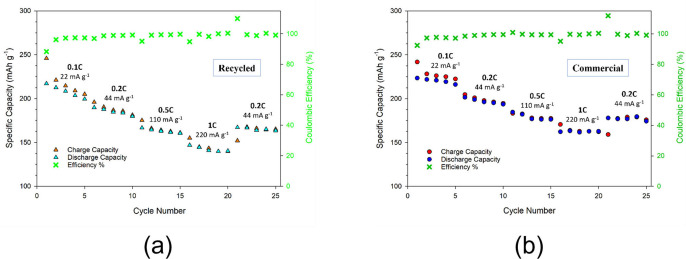
Comparison of the rate performances between (a) recycled and (b) commercial LNCO cathodes at different C rates (0.1 C: *I*=22 mA g^−1^; 0.2 C: *I*=44 mA g^−1^; 0.5 C: *I*=110 mA g^−1^; 1 C: *I*=220 mA g^−1^, and 0.2 C: *I*=44 mA g^−1^ as recovery)

The active material obtained through this recycling process was characterized from an electrochemical point of view by tests of galvanostatic cycling at room temperature in order to determine the specific capacity delivered upon charge and discharge and the corresponding cathode rate capability. Figure 11 compares the functional performances of two cells, one including the recycled cathode (part a), and the other obtained from the commercial active material (part b) as a reference. Both the cells were loaded with a solution of 1 m LiPF_6_ in ethylene carbonate/dimethyl carbonate (EC/DMC, 50 : 50 vol %) as electrolyte and cycled from 2.7 to 4.5 V at different C rates (0.1, 0.2, 0.5, 1 C). As noticeable from Figure 11, quite comparable cycling performance are obtained, with capacity values very close to the theoretical one. In case of lower current density, coulombic efficiency very close to 1 and specific capacities of approximately 200 and 190 mAh g^−1^ were delivered respectively by the cells with the commercial cathode and recycled one.

## Conclusions

A deep eutectic solvent (DES)‐based green solvometallurgical process for cathodes recycling from spent lithium‐ion batteries (LIBs) was demonstrated for three active materials: LiMn_2_O_4_ (LMO), LiNi_0.5_Mn_1.5_O_4_ (LNMO), and LiNi_0.8_Co_0.2_O_2_ (LNCO). Two DESs were compared, namely choline chloride–lactic acid (ChCl:LA) and choline chloride–glycerol (ChCl:Gly), differing for the system acidity. For the first time, to our best knowledge, it was showed that the DES with lower pH, ChCl:LA, can easily extract critical metals as Li, Mn, Ni, and Co from cathode active materials, achieving leaching yields of 100 %. If the material concentration in DES is properly optimized, ChCl:LA leads to full metal oxide dissolution in significantly shorter times and lower temperatures than those to date reported in the literature.

In the case of LNCO, Li, Ni, and Co were easily recovered with a high rate (>85 %) through safe and green precipitation approaches as useful precursors for the re‐synthesis of the starting active materials (for example LiPO_3_ and Ni/Co oxalate) or for other valuable compounds. In addition, by using solid oxalic acid as the precipitant agent, the leaching DES may be easily regenerated after the separation of Ni and Co and employed for at least three more times without any significant loss of efficiency, and accumulating Li, which can be recovered all at once as the final step. The proposed all‐green and circular strategy is currently a proof‐of‐concept that has great potential of feasibility also in case of more complex active materials (e. g., in case of Ni/Mn/Co‐based systems). After proper validation in real systems, it will be a step forward more sustainable processes of end‐of‐life LIB recycling.

## Experimental Section

### Starting materials

DESs were prepared starting from commercial compounds, namely ChCl, Gly, and LA (Sigma‐Aldrich). Three different active materials for LIB cathode were investigated, which are LMO, LNCO, and LNMO, purchased from Merck and Sigma‐Aldrich, respectively.

### Metal extraction procedure

Two different DESs were prepared: (1) ChCl:Gly, and (2) ChCl:LA. In both cases the two components were mixed in a molar ratio 2 : 1, heated at 50 °C under magnetic stirring until complete dissolution, and degassed for 15 min under N_2_ atmosphere before the use.

The cathode active material was dissolved in the DES and kept at a constant temperature (in the range 20–170 °C) for a given time (5–24 h) in a thermostatic oil bath under continuous stirring. After the treatment, the eventual undissolved residue was filtered, and the leachate was analyzed for quantification. In the specific case of LNCO, the metal leaching procedure was optimized by means of an DoE approach, as better described in the following. The leaching efficiency was determined using Equation 2:

(1) $$Leaching\;\left[\%\right]=\frac{C_{M,f}\times V_{s}}{m_{M}}$$

where *C*_M,f_ is the metal concentration in the leaching solution [mg L^−1^], *V*_s_ is the initial leaching solution volume [L], and *m*_M_ the amount of metal in the pristine cathode [mg].

### Metal recovery after leaching

The protocol of metal recovery as valuable final products has been developed for LNCO. In this specific case, Ni and Co dissolved in the leaching solution were recovered through precipitation with OA to obtain mixed Ni/Co oxalate. This step was carried out by two different approaches: (i) by using an OA‐saturated aqueous solution (0.25 m), or (ii) by using pure OA. In the first case, a volume containing an excess of OA (OA/Ni−Co molar ratio: 1.5 : 1) was added to the filtrate, resulting in a change of the solution color from blue to green. Such a solution was stirred at 40 °C for different times (30 min, 1 h, and 4 h) and then centrifuged twice at 6000 rpm for 30 min to collect the light‐green solid precipitate, which was finally characterized. The resulting solution was then dried by a rotary evaporator to recover the initial DES, including Li ions. In the second route, an excess of solid OA (OA/Ni−Co molar ratio: 1.5 : 1) was added to the leaching solution, which was then stirred at 40 °C for 30 min and centrifuged twice at 6000 rpm for 30 min to collect the pale green precipitate. In both cases, the green solid was characterized both as obtained and after calcination. Specifically, the calcination step was carried out at 800 °C for 16 h by heating at 10° min^−1^. The sample was then spontaneously cooled down to room temperature in oven.

After the Ni and Co separation, Li was finally recovered by treating the solution with an excess of H_3_PO_4_ 0.5 m aqueous solution, which was then heated under continuous stirring overnight at 100 °C to obtain a viscous gel with white precipitated crystals. This product was dried at 300 °C in a furnace, and subsequently calcined at 750 °C for 12 h to obtain LiPO_3_.

### Synthesis of cathode materials from the recovered metals

In the specific case of LiNi_0.8_Co_0.2_O_2_, the active material was re‐produced using a solid‐state reaction between Ni−Co oxalate and LiPO_3_, both recovered as described before. The compounds were previously mixed in a mortar, then treated at 550 °C for 12 h and finally at 750 °C in air overnight.

### Cathode preparation and cell assembly

The cathode slurry was prepared by using 70 wt% of active material (LNCO), 20 % conductive carbon black (Imerys, Ensaco 350P) and 10 % binder (PVDF). The solid content of all slurries was kept between 24 and 26 wt%. LNCO powder and carbon were mixed in zirconia jars by a planetary ball mill at 150 rpm for 10 min, followed by a 5 min break and another 10 min of milling. Subsequently, the polymeric binder was added and mixed with an analogue procedure. The as‐prepared mixed powder was dispersed in *N*‐methylpyrrolidone (NMP) to obtain the slurry, which was casted onto a carbon‐coated Al foil using a doctor blade with a wet thickness of 300 μm. The casted slurry was dried under vacuum at 104 °C for 14 h to avoid moisture and oxygen contamination. The cathode was finally cut into 2 cm^2^ disks and stored in a glovebox (MBraum, O_2_, H_2_O<0.5 ppm) before the electrochemical measurements.

### PVDF and Al stability in DES

In order to check compatibility with ChCl/LA of the other cathode components, namely PVDF (binder), aluminum, and copper (current collectors), 2 mg of polymer and metals were dispersed in 7.5 mL of DES and treated at 105 °C for 5 h under vigorous magnetic stirring.

### Characterization methods

ICP‐OES was performed by a Thermo Scientific CAP 7400 Duo, equipped with a quartz torch, a charge injection detector and a Cetac ASX‐560 autosampler. Each element was quantified by choosing 1 : 50 as dilution factor. The quantification was carried out in the radial mode by an external standard calibration curve. ICP‐grade standards 1000 mg L^−1^ (Merck) were diluted to 1–5–10 mg L^−1^ and then acidified to a final concentration of 1 % nitric acid (from ultrapure 65 % HNO_3_, Merck). The measurements conditions were as in the following: nebulization gas flow: 0.5 L min^−1^; power RF: 1150 W; cooling gas flow: 12 L min^−1^; auxiliary gas flow: 0.5 L min^−1^; peristaltic pump speed: 50 rpm; frequency: 500 Hz; intake flow: 1.5 L min^−1^. The reported quantitative data are the average of at least two replicates.

DSC analyses were performed with a Q2000 instrument (TA Instruments, USA) by heating the samples (about 20 mg) from −80 to 150 °C at 5 °C min^−1^, under N_2_ atmosphere in Al crucibles sealed in the glovebox.

For FTIR spectroscopy, a Nicolet FT‐IR iS10 spectrometer (Nicolet, Madison, WI, USA) equipped with attenuated total reflectance (ATR) sampling accessory (Smart iTR with diamond plate) was used. 32 scans in the 4000–600 cm^−1^ range at 4 cm^−1^ resolution were coadded. Well‐ground powder samples were used, and spectra were obtained after pressing the sample towards the ATR diamond crystal at room temperature (20 °C). Peaks wavenumbers were attributed by using the “Find peaks” function of the OMNIC™ Spectra Software.

Powder XRD was carried out by using a D8 Advance diffractometer (Bruker). SEM and EDX were performed using a Tescan Mira3XMU microscope operated at 20 kV and equipped with an EDAX EDS microanalysis system. The samples were coated with a carbon thin film using a Cressington 208 carbon coater.

UV/Vis absorption spectra were collected by means of a UV/Visible/NIR spectrophotometer Jasco V750 in absorbance mode between 900 and 250 nm with scan speed of 400 nm min^−1^ and data interval of 0.5 nm. The liquid setup with double cuvette (containing water as a reference and the sample) was used.

All electrochemical measurements were performed in a coin cell type (CR2032 ‐ MTI Corp.) assembled in an Ar‐filled glovebox (H_2_O and O_2_<0.5 ppm). Metallic Li was used as counter electrode. Electrodes were separated with a Whatman glass fiber separator, imbibed by the liquid electrolyte, consisting of solution 1 m LiPF_6_ in EC/DMC (50 : 50 vol %) (provided by Sigma–Aldrich) (120–150 μL). Potentiostatic electrochemical impedance spectroscopy (PEIS) was performed on the cells using a battery tester Bio‐Logic BCS‐810.

The cells were galvanostatically cycled at room temperature from 2.7 to 4.5 V at various C rates (0.1, 0.2, 0.5, 1 C). A theoretical capacity of 219 mAh g^−1^ was considered.

## Conflict of interest

The authors declare no conflict of interest.

## Supporting information

As a service to our authors and readers, this journal provides supporting information supplied by the authors. Such materials are peer reviewed and may be re‐organized for online delivery, but are not copy‐edited or typeset. Technical support issues arising from supporting information (other than missing files) should be addressed to the authors.

Supporting Information
