# Supplementary material for: Cathode Active Material Recycling from Spent Lithium Batteries: A Green (Circular) Approach Based on Deep Eutectic Solvents
Source: ChemSusChem. 2021 Dec 23;15(2):e202102080. doi: 10.1002/cssc.202102080 (PMC9305586; doi:10.1002/cssc.202102080)
Supplement: Supplementary file 1 — Supporting Information [file CSSC-15-0-s001.pdf]

ChemSusChem

Supporting Information

Cathode Active Material Recycling from Spent Lithium Batteries: A Green (Circular) Approach Based on Deep Eutectic Solvents

Riccardo Morina[+], Daniele Callegari[+], Daniele Merli, Giancarla Alberti, Piercarlo Mustarelli, and Eliana Quartarone*© 2021 The Authors. ChemSusChem published by Wiley-VCH GmbH. This is an open access article under the terms of the Creative Commons Attribution License, which permits use, distribution and reproduction in any medium, provided the original work is properly cited.

Author Contributions

R.M. experiments:Equal
D.C. experiments:Equal
D.M. Methodology:Supporting
G.A. Methodology:Supporting
P.M. Writing – review & editing:Supporting

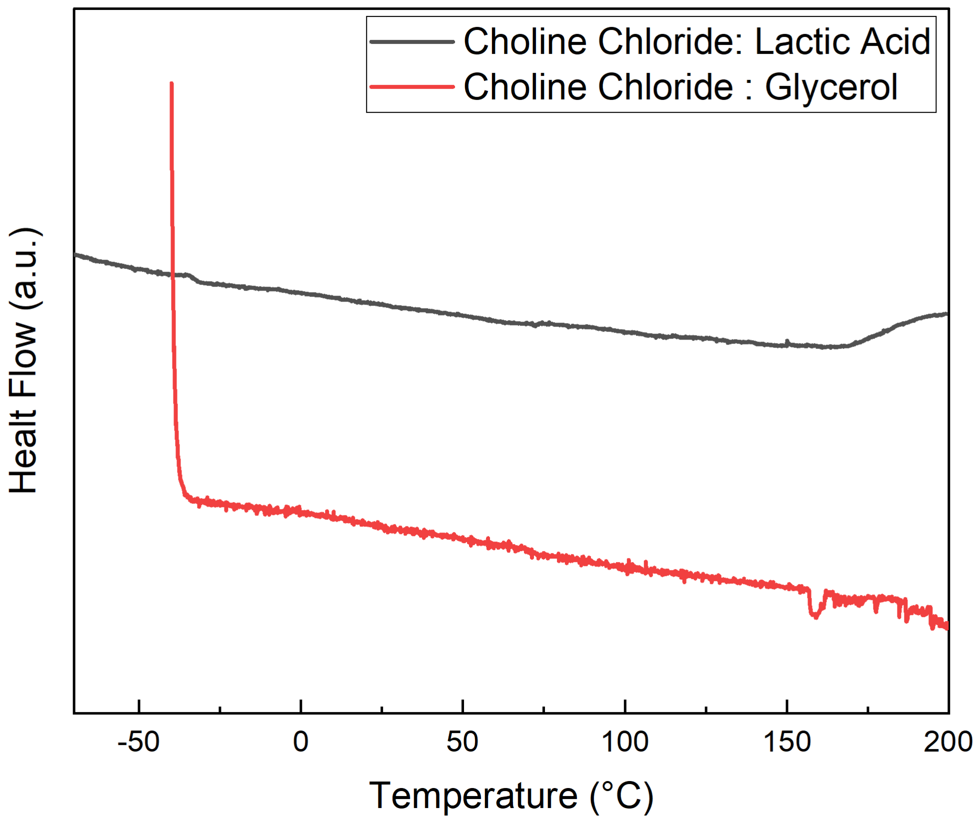

Figure S1: Differential Scanning Calorimetry (DSC) plots of the investigated DESs: ChCl:LA (black line) and ChCl:Gly (red line).

				A			B		
**Experiment**	**T (°C)**	**Time (h)**	**DES (g)**	**Co %**	**Li %**	**Ni %**	**Co %**	**Li %**	**Ni %**
**1**	**50**	**5**	**2.5**	33.0	40.6	30.7	37.6	52.5	34.4
**2**	**105**	**5**	**2.5**	87.8	72.4	86.5	79.5	68.3	78.1
**3**	**50**	**24**	**2.5**	51.5	58.5	51.1	52.2	66.3	50.3
**4**	**105**	**24**	**2.5**	88.2	74.0	87.5	1.5	71.1	1.5
**5**	**50**	**5**	**7.5**	62.4	62.4	62.1	58.3	59.5	57.1
**6**	**105**	**5**	**7.5**	105.9	138.8	106.1	100.0	111.7	97.8
**7**	**50**	**24**	**7.5**	70.6	68.1	70.8	71.0	72.6	71.4
**8**	**105**	**24**	**7.5**	100.1	131.9	104.6	88.1	87.2	84.1

				**A**			**B**		
**Experiment**	**T (°C)**	**Time (h)**	**DES (g)**	**Co %**	**Li %**	**Ni %**	**Co %**	**Li %**	**Ni %**
**9**	**77,5**	**14,5**	**5**	64,7	67,3	64,9	72,3	66,6	71,0

Table S1: Leaching efficiency quantitative chemical analysis of Li, Co and Ni obtained by ICP-OES measurements used for the Experimental Design in LNCO.

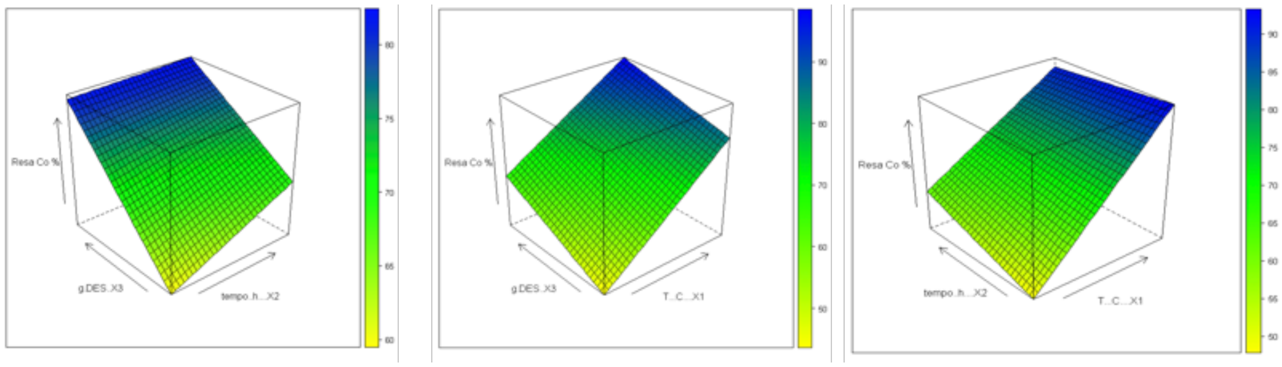

(i)

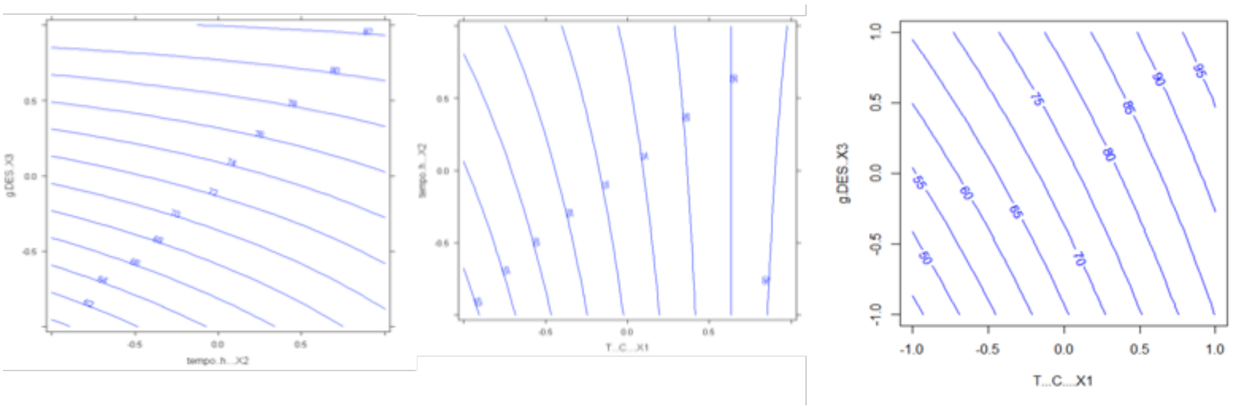

(ii)

Yield, Co (%)	Yield, Co (%) fit
33,0	39,0
87,8	76,2
51,5	52,5
88,2	89,7
62,4	64,9
105,9	102,2
70,6	61,9
100,1	99,2
37,6	39,0
79,5	76,2
52,2	52,5
85,0	89,7
58,3	64,9
100,0	102,2
71,0	61,9
88,1	99,2

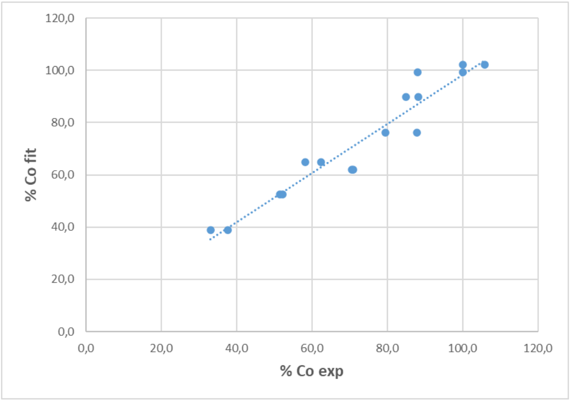

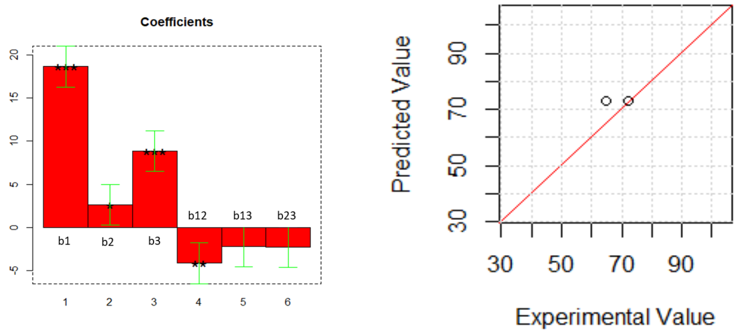

(iii)

Figure S2a: Results of the Experimental Design (DOE) for Co: Response Surface (i); Contour Plots Rate versus time and rate versus temperature (ii); yield and DOE coefficients (iii).

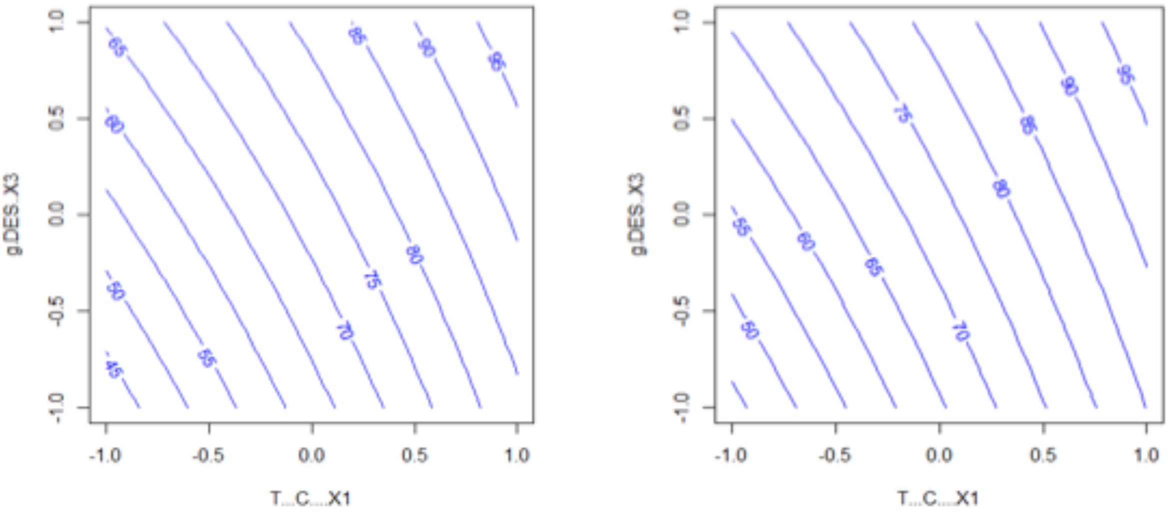

(i)

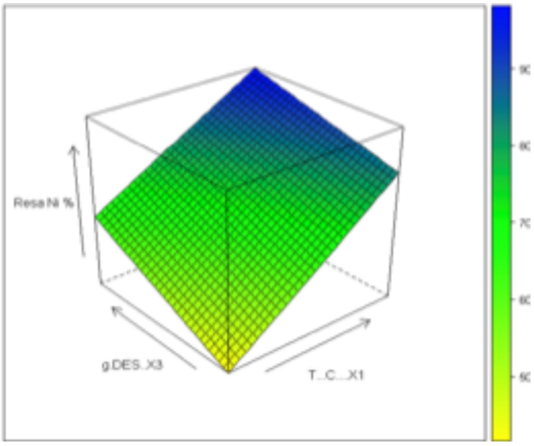

(ii)

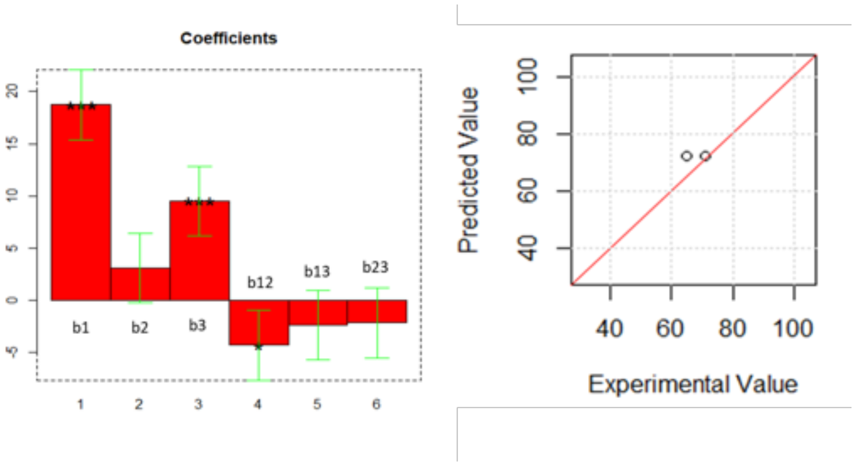

(iii)

Figure S2b: Results of the Experimental Design (DOE) for Ni: Contour Plots Rate versus temperature (i) Response Surface (ii); yield and DOE coefficients (iii).

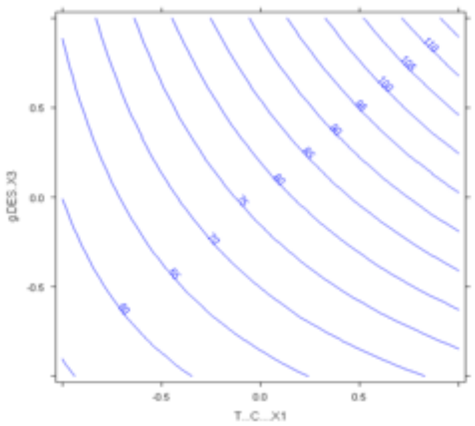

(i)

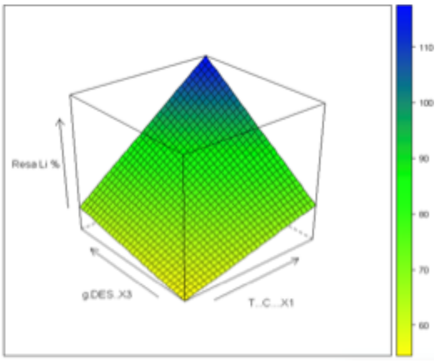

(ii)

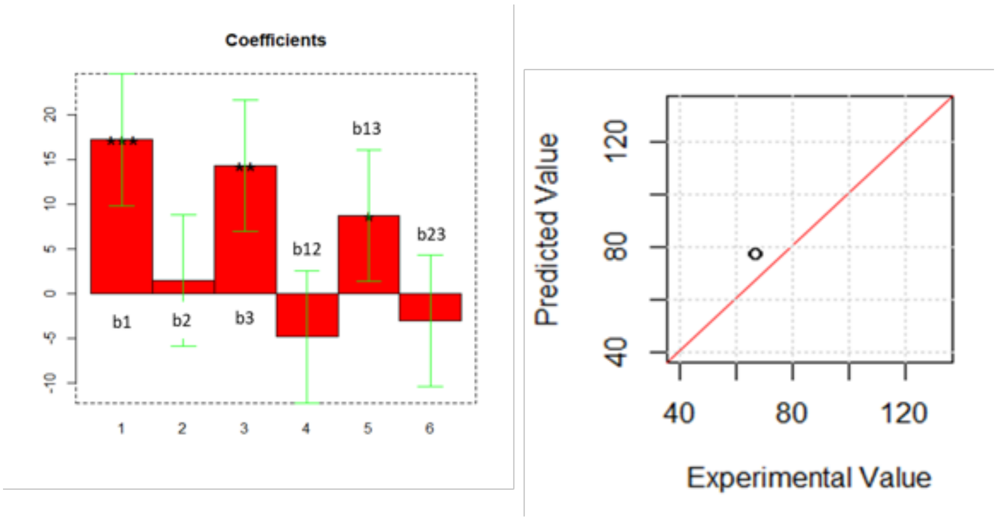

(iii)

Figure S2c: Results of the Experimental Design (DOE) for Li: Contour Plots Rate versus temperature (i) Response Surface (ii); yield and DOE coefficients (iii).

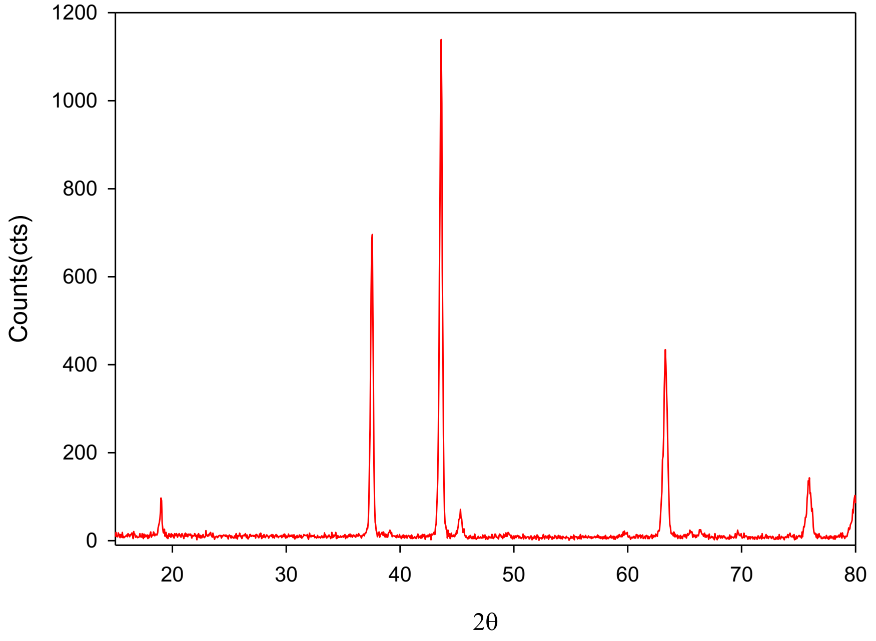

(a)

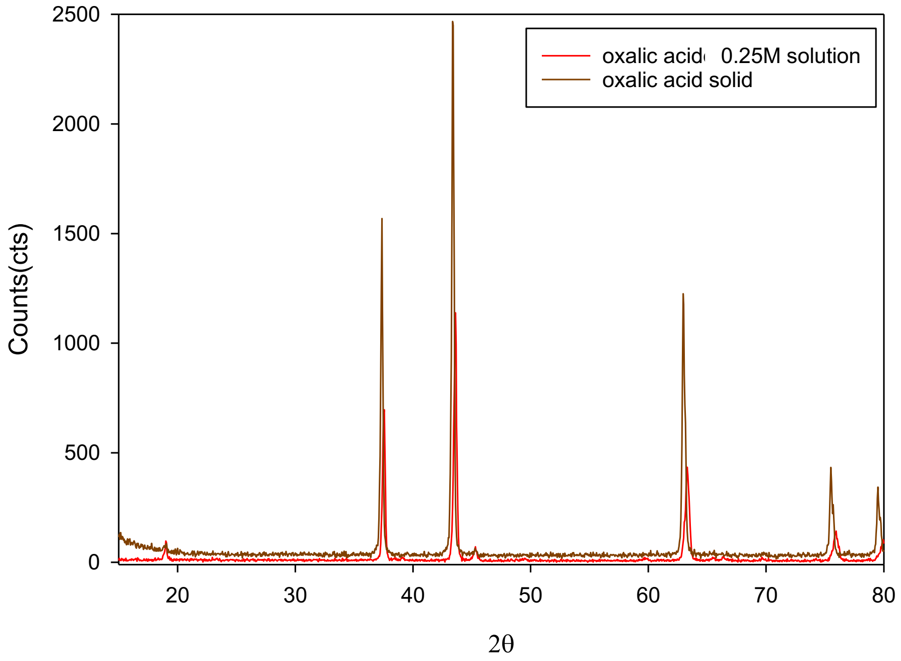

(b)

Figure S3: (a) XRD pattern of Nickel oxalate recovered by precipitating Ni and Co with solid oxalic acid; (b) comparison of the XRD patterns for the Ni and Co oxide obtained by calcinating the corresponding oxalate salt recovered with oxalic acid, both in aqueous solution (red line) or pure solid (black line).

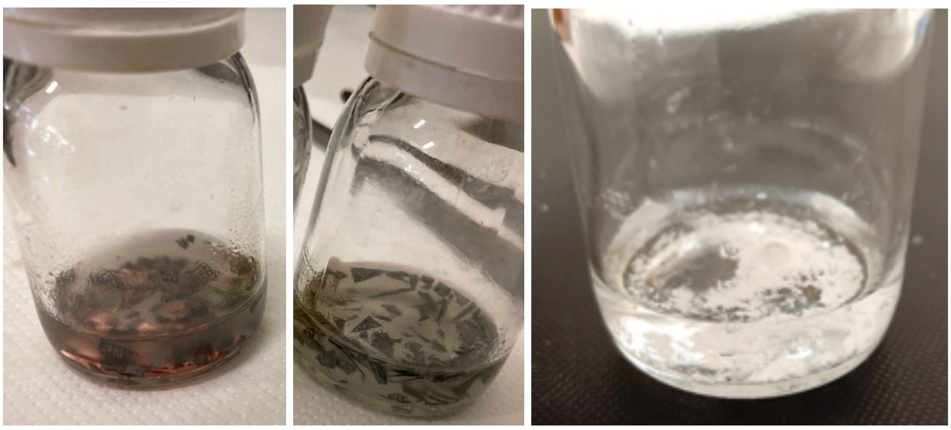

(a) (b) (c)

Figure S4: Images of current collectors in the selected DES, (a) Cu and Al; (b) Al; (c) PVDF binder treated at 105°C for 5 hours.
